# Optimization of ribosome profiling using low-input brain tissue from fragile X syndrome model mice

**DOI:** 10.1093/nar/gky1292

**Published:** 2018-12-24

**Authors:** Botao Liu, Gemma Molinaro, Huan Shu, Emily E Stackpole, Kimberly M Huber, Joel D Richter

**Affiliations:** 1 Program in Molecular Medicine, University of Massachusetts Medical School, Worcester, MA 01605, USA; 2 Department of Neuroscience, University of Texas Southwestern Medical Center, Dallas, TX 75390, USA

## Abstract

Dysregulated protein synthesis is a major underlying cause of many neurodevelopmental diseases including fragile X syndrome. In order to capture subtle but biologically significant differences in translation in these disorders, a robust technique is required. One powerful tool to study translational control is ribosome profiling, which is based on deep sequencing of mRNA fragments protected from ribonuclease (RNase) digestion by ribosomes. However, this approach has been mainly applied to rapidly dividing cells where translation is active and large amounts of starting material are readily available. The application of ribosome profiling to low-input brain tissue where translation is modest and gene expression changes between genotypes are expected to be small has not been carefully evaluated. Using hippocampal tissue from wide type and fragile X mental retardation 1 (*Fmr1*) knockout mice, we show that variable RNase digestion can lead to significant sample batch effects. We also establish GC content and ribosome footprint length as quality control metrics for RNase digestion. We performed RNase titration experiments for low-input samples to identify optimal conditions for this critical step that is often improperly conducted. Our data reveal that optimal RNase digestion is essential to ensure high quality and reproducibility of ribosome profiling for low-input brain tissue.

## INTRODUCTION

Regulated protein synthesis in the synapto-dendritic compartment of neurons is essential to establish and maintain the brain circuitry that underlies higher cognitive function (1). Dysregulation of local protein synthesis is known to cause several neurological disorders including autism and fragile X syndrome (FXS) (2). FXS is caused by a CGG repeat expansion in the 5′ untranslated region (UTR) of fragile X mental retardation 1 (*FMR1*), which leads to epigenetic silencing and loss of its protein product, fragile X mental retardation protein (FMRP). FMRP is a complex RNA binding protein that mediates gene expression at multiple levels, but acts most prominently as an inhibitor of translation (3). In *Fmr1*-deficient mouse brain (hippocampus), general protein synthesis is ∼15% greater than in wild type (4), which is thought to be sufficient for driving several autism and FXS-like characteristics such as learning and memory deficits and synaptic deficiencies (5). How a relatively modest change of protein synthesis can be responsible for large phenotypic alterations is at last partly explained by signal amplification resulting from kinase cascades that emanate from the synapse (6). Additionally, FXS pathophysiologies may also result from low abundance mRNAs that undergo large changes in translation or from the aggregation of small changes in translation of numerous mRNAs. Moreover, in contrast to most cells, neurons are particularly vulnerable to disruption of dosage and dynamics of RNA-binding proteins involved in RNA metabolism (7,8). These unique features plus the fact that the brain is composed of multiple cell types pose significant challenges for reliably detecting differences in translation between normal versus disease conditions.

Ribosome profiling is a genome-wide approach to monitor protein synthesis by sequencing ribosome protected mRNA fragments (RPFs) after ribonuclease (RNase) digestion (9). Ribosome profiling has been widely utilized to study various aspects of mRNA translation, including translational efficiency regulation (10), alternative initiation (11), elongation pausing (12), co-translational protein folding (13) and codon usage (14). Since its initial development in yeast (15), ribosome profiling has been rapidly applied to many other mitotic cell systems (16) and more recently post-mitotic somatic tissues (17). For the central nervous system, application of ribosome profiling has been mostly limited to the whole brain (18) or pooled brain regions (19,20). Given the complex cell composition and circuit organization of the brain, there is an urgent need to apply ribosome profiling to more defined brain regions or specific cell types where biological material is limited.

Although ribosome profiling is a very powerful technique to study mRNA translation with high resolution, it can suffer from biases introduced by translational inhibitor pre-treatment (21), RNase digestion (22), and library preparation (23). Biases can also be introduced bioinformatically and computationally (24–26). These issues have led to inconsistent results from different laboratories and contradictory findings, especially for elongation pausing and codon usage studies that rely on the localized ribosome footprint densities (27,28). Therefore, accurate methods that minimize technical biases and reveal the true biological signal are necessary in analyses of the brain, especially when considering neurological disease models such as FXS where translational changes are modest between genotypes.

We used the study of translational dysregulation in FXS model mice as a proof-of-principle to address the major issues of ribosome profiling for low-input brain samples where the differences between mouse genotypes (i.e. WT and *Fmr1* KO) are expected to be small. Here, for the first time, we describe significant sample batch effects caused by the inconsistency of RNase digestion, which may help explain contradictory findings (29). This type of batch effect can be indirectly quantified by the GC content and length of ribosome footprints. We establish a workflow of RNase titration experiments for low-input samples to determine the optimal enzyme concentration. We proposed a better practice of this critical RNase digestion step that is often improperly conducted. In summary, our data reveal that optimal RNase digestion is essential to ensure high quality and reproducibility of ribosome profiling especially for low-input brain tissue.

## MATERIALS AND METHODS

### Mouse brain tissue collection

Mouse protocols were reviewed and approved by the institutional animal care and use committee (IACUC), and all colonies were maintained following animal research guidelines. FXS affects both males and females, but females often have milder symptoms and a lower rate of cognitive impairment than males (30). Thus, historically, most preclinical studies have employed male mice, including electrophysiology (31) and expression profiling experiments (32). Therefore, to compare our results to previous studies, we chose to proceed with male mice. Acute hippocampal brain slices were prepared from P28–35 C57BL/6J wild-type or *Fmr1* KO male mice littermates (6–8 mice per batch) as previously described (33). For intact brain tissues, cortical or hippocampal tissues were collected from P35 C57BL/6 wild-type male mice as previously described (32), snap-frozen in liquid nitrogen, and stored at −80°C.

### iPSC differentiation and collection

Human induced pluripotent stem cell (iPSC) colonies were maintained in mTeSR1 medium (STEMCELL Technologies). Neural progenitor cells (NPCs) were derived from iPSCs using STEMdiff Neuron Differentiation Kit (STEMCELL Technologies) and maintained in STEMdiff Neural Progenitor Medium (STEMCELL Technologies). For the neural differentiation, NPCs were plated at 2 × 10^4^ cells/cm^2^ onto poly-l-ornithine/laminin coated 15-cm dishes in neural differentiation medium [Neurobasal Medium (Gibco), 1× N-2 supplement (Gibco), 1× B-27 supplement (Gibco), 1× GlutaMAX (Gibco), 0.2 μM l-ascorbic acid (Sigma), 1 μM cAMP (Sigma), 10 ng/ml BDNF (Peprotech), 10 ng/ml GDNF (Peprotech)] supplemented with 0.1 μM Compound E (Millipore) and 5 μM ROCK inhibitor (Millipore). Neural cultures were maintained in neural differentiation medium for 1 month before collection. For the iPSC sample collection, cyclohexmide was added to the medium to a final concentration of 100 μg/ml and cells were incubated at 37°C for 1 min. The cells were subsequently washed with ice-cold PBS containing 100 μg/ml CHX, collected by scraping from the dish, pelleted by centrifugation at 15 000g 4°C for 3 min, snap-frozen in liquid nitrogen, and immediately stored at −80°C.

### Sucrose gradients of intact brain tissues

Frozen cortices or hippocampi from P35 WT male mice were thawed in ice-cold homogenization buffer [20 mM Tris–HCl pH 7.4, 5 mM MgCl_2_, 100 mM KCl, 1 mM DTT, 100 μg/ml CHX, 25 U/ml Turbo DNaseI (Ambion, #AM2238), 1× EDTA-free protease inhibitor (Roche), avoid detergent, in nuclease-free water] on ice for 10 min. Wide orifice tips were used to transfer tissue to a pre-chilled detergent-free Dounce homogenizer. Tissues were slowly homogenized by hand (10 strokes of loose pestle A, and 10 strokes of tight pestle B). Homogenates were carefully transferred to clean 1.5 ml tubes with clean glass Pasteur pipets and bulbs. Homogenates were clarified by centrifugation at 2000g 4°C for 10 min. The supernatants were collected and clarified again by centrifugation at 20 000g 4°C for 10 min. The supernatants were collected and the amounts of nucleic acid were measured with Nanodrop (*A*_260_ units). The homogenates were adjusted and diluted with homogenization buffer to 0.3 ml for each gradient. The samples were digested with RNase A (Sigma, # R4875; or Ambion, #AM2270 for the titration), RNAse T1 (Thermo Fisher Scientific, #EN0542), or RNase I (Ambion, #AM2294) as indicated in the figure legends. The digestion reactions were stopped by chilling on ice and adding 50 U SUPERase In RNase inhibitor (Ambion, #AM2694). 1% NP-40 was added to the homogenates and incubated on ice for 10 min. Digested homogenates were clarified again by centrifugation at 20 000g 4°C for 10 min. The supernatants were applied to 10%-50% sucrose gradients prepared in 1× polysome buffer (20 mM Tris–HCl pH 7.4, 5 mM MgCl_2_, 100 mM KCl, 1 mM DTT, 100 μg/ml CHX in nuclease-free water). After the ultracentrifugation in a SW41Ti rotor (Beckman Coulter) at 35 000 rpm (average 151 263g) 4°C for 2.5 h, gradients were fractionated at 1.5 ml/min and 12 s collection interval through a fractionation system (Brandel) that continually monitored *A*_260_ values. Monosome fractions were identified, pooled, and extracted with TRIzol LS (Invitrogen, #10296028).

### Sucrose gradients of hippocampal slices

Frozen hippocampal slices were thawed in ice-cold homogenization buffer on ice for 5 min. Wide orifice tips were used to transfer slices to a pre-chilled detergent-free Dounce homogenizer. Tissues were slowly homogenized by hand (20 strokes of loose pestle A, and 20 strokes of tight pestle B). Homogenates were carefully transferred to clean 1.5 ml tubes with clean glass Pasteur pipets and bulbs. 1% NP-40 was added to the homogenates and incubated on ice for 10 min. Homogenates were clarified by centrifugation at 2000g 4°C for 10 min. The supernatants were collected and clarified again by centrifugation at 20 000g 4°C for 10min. The supernatants were collected and the amounts of nucleic acid were measured with Nanodrop (*A*_260_ units). The samples were digested with 100ng RNase A (Sigma, # R4875) and 60U RNase T1 (Thermo Fisher Scientific, #EN0542) per *A*_260_ at 25°C for 30 min and stopped by chilling on ice and adding 50 U SUPERase In RNase inhibitor (Ambion, #AM2694). Digested lysates were applied to 10–50% sucrose gradients similarly as the intact brain tissues above.

For each new lot of RNase, we recommend performing a quality control test for the enzymatic activity. For instance, similar RNase titration experiments with a new lot of enzyme could be performed as in Figure 5. If the new lot of RNase also causes noticeable monosome disassembly at Conc.5, it has comparable enzymatic activity and the optimized digestion condition determined by the titration test could be used with the new lot.

### Sucrose gradients of iPSC samples

Frozen cell pellets were thawed in ice-cold polysome lysis buffer [20 mM Tris–HCl pH 7.4, 5 mM MgCl_2_, 100 mM KCl, 1 mM DTT, 100 μg/ml CHX, 25 U/ml Turbo DNaseI (Ambion, #AM2238), 1× EDTA-free protease inhibitor (Roche), 1% NP-40 in nuclease-free water] and lysed by trituration through a 25-G need for 10 times. The remaining steps are the same as the hippocampal slices above. See Supplementary Table S1 for the summary of all samples used in this paper.

### Ribosome profiling

Ribosome profiling libraries were prepared following the published protocols (34). Briefly, rRNA was depleted from the purified monosomal RNA samples with RiboZero (Illumina, #MRZG12324). Remaining RNA samples were separated on a 15% TBU gel (National Diagnostics, #EC-833) and the ribosome footprints were size-selected between the 26 and 34nt markers. RNA was eluted from the crushed gel pieces in RNA elution buffer (300 mM NaOAc pH 5.5, 1 mM EDTA, 0.25% SDS) at room temperature overnight, filtered with Spin-X Centrifuge Tube Filters (Corning, #8162) and precipitated with equal volume of isopropanol. Recovered RNA was dephosphorylated with T4 Polynucleotide Kinase (NEB, #M0201S) and ligated with preadenylated adaptor in miRCat^®^-33 Conversion Oligos Pack (IDT) using T4RNL2Tr.K227Q ligase (NEB, #M0351L). Reverse transcription (RT) was performed with primers containing 5nt-barcodes and 8nt-unique molecular identifiers (UMIs) and SuperScript III (Invitrogen, #18080-044) in 1X first-strand buffer without MgCl_2_ (50 mM Tris–HCl, pH 8.3, 75 mM KCl). RT products were separated on a 10% TBU gel and the 130–140nt region was selected. cDNA was eluted in DNA elution buffer (10 mM Tris pH 8.0, 300 mM NaCl, 1 mM EDTA) at room temperature overnight, filtered and precipitated with isopropanol. Purified cDNA was circularized with CircLigase (Epicentre, #CL4115K). Except for the RNase titration samples, cDNA derived from remaining rRNA was hybridized to biotin-labelled antisense probes (IDT) and further depleted with Dynabeads MyOne Streptavidin C1 (Invitrogen, #65001). The sequences and ratio of antisense probes need to be determined based on the RNase digestion condition used. Optimal PCR cycle number was determined empirically by test PCR reactions with titrated cycle numbers. Final PCR amplification was performed with KAPA Library Amplification Kit (Kapa Biosystems, #KK2611) and 180–190 bp products were size-selected on an 8% TBE gel. DNA was eluted in DNA elution buffer, filtered, and precipitated with isopropanol. The final library DNA was purified with AMPure XP beads (Beckman Coulter, #A63880). Oligos used for the library preparation are listed in Supplementary Table S2.

The size distributions of final libraries were measured by Fragment Analyzer (Advanced Analytical, performed by Molecular Biology Core Labs at UMMS). The concentrations were quantified with KAPA Library Quantification Kit (Kapa Biosystems, #KK4835). Libraries were pooled with equal molar ratios, denatured, diluted, and sequenced with NextSeq 500/550 High Output Kit v2 (Illumina, #FC-404-2005) on a Nextseq500 sequencer (Illumina).

For low-input samples, we recommend adding 10 mM MgCl_2_ to the isopropanol precipitations of short RNA/cDNA fragments and incubating the reactions at −20°C overnight to increase the yields. In our hands, as compared to other popular brands, the combination of 20 μg glycogen carrier (Ambion, #AM9510) and 1.5 ml tubes (LPS, # L250901) for the precipitation improves the efficiencies of pellet formation and nucleic acid recovery significantly after centrifugation at 20 000g 4°C for at least 30 min.

### Read mapping and quality control

Individual samples were separated from the raw fastq files based on the barcode sequences. Adaptor sequences (TGGAATTCTCGGGTGCCAAGGAGATCGGAAGAGCGGTTCAGCAGGAATGCCGAGACCG) were removed with cutadapt (1.7.1). Trimmed fastq files were uploaded to the Dolphin platform (https://www.umassmed.edu/biocore/introducing-dolphin/) at the UMMS Bioinformatics Core for the mapping steps. Trimmed reads were quality filtered with Trimmomatic (0.32) and mapped to the mouse rRNA and then tRNA references with Bowtie2 (2.1.0). Unmapped reads were next mapped to the mm10 mouse genome with Tophat2 (2.0.9). Reads mapped to >1 loci of the genome were classified as ‘multimapped’ reads and discarded. PCR duplicates were marked based on the UMI sequences with custom scripts and only uniquely mapped reads without duplicates were retained with samtools (0.0.19) for the downstream analyses.

Reads mapped to 5′ untranslated region (5′UTR), coding sequences (CDS), and 3′UTR regions were counted after intersecting the bam files with bed annotation files (USCS genome browser) using bedtools (2.22.0). Sequences of reads uniquely mapped to CDS were extracted with samtools (0.0.19). The nucleotide composition and the distribution of RPF length were calculated with custom scripts. The mean GC percentages within 10–20nt window for RPF or 10–65nt window for mRNA were calculated for each sample. The peak of RPF length distribution was selected as the representative length for the scatter plots. P-site offsets and frame preference were calculated with plastid (0.4.8). Counts at each nucleotide position were extracted using P-sites of RPFs, normalized to the library size, averaged across replicates, and plotted along mRNA positions with custom scripts.

### Differential gene expression analysis

Cleaned bam files of uniquely mapped RPFs were converted to fastq files with bedtools. Gene expression was quantified with RSEM (1.2.11) using the cleaned fastq files and Refseq (V69) mouse CDS without the first and last 30nt to avoid the translation initiation and termination peaks. Genes were filtered with minimum 5 average reads across all replicates. Read counts were transformed with regularized logarithm on the log_2_ scale and normalized with respect to library size. Scatter plots with rlog transformed counts were used to evaluate the correlations among samples. Top 1000 most variable genes were selected for PCA analysis using the ‘prcomp’ function of ‘stats’ package and the results were plotted with ‘ggbiplot’ package. Counts were batch-corrected with the ‘Combat’ function in ‘sva’ (3.24.4) using a full model matrix. Differential gene expression analysis was performed with batch-corrected counts and DESeq2 with a 0.05 cut-off for adjusted *P*-value. Based on the Refseq mouse reference transcriptome, the most abundant transcript isoform estimated by RSEM with Batch1_WT1 RNA-seq data was picked as the representative transcript for each gene. Then, GC contents of the annotated sequences for various mRNA regions were calculated across the transcriptome and plotted in Figure 2D. In contrast, GC contents based on all the RNA-seq reads mapped to CDS were calculated in Figure 2E, so reads from well-expressed mRNAs are over- represented.

## RESULTS

### Sample batch effects dramatically compromise the power of ribosome profiling

Ribosome profiling was initially established by Ingolia *et al.* in yeast with RNase I, a nucleotide-indiscriminate endoribonuclease that preferentially hydrolyzes single-stranded RNA, to generate loaded 80S monomers (15). Although RNase I generates high-resolution yeast ribosome profiling data, it causes significant or even complete disassembly of mammalian ribosomes under similar conditions (22) (Supplementary Figure S1). Because actively translating ribosomes in post-mitotic neuronal tissue are substantially fewer than in mitotic cells, we decided to use a cocktail of RNase A and T1 that maintains the integrity of monosomes for mammalian samples (35) (Supplementary Figure S1).

Most ribosome profiling studies as well as a commercially available TruSeq Ribo Profile kit (Illumina) adjust the amounts of RNase to digest the polysomes based on the amounts of nucleic acids in the lysate typically estimated by the total *A*_260_ units. For example, TruSeq Ribo Profile kit suggests to digest lysate with 5 units TruSeq Ribo Profile Nuclease per *A*_260_ unit at 25°C for 45 min. To determine the optimal RNase concentration for neuronal tissues, we performed an RNase titration experiment with mouse cortical samples and used the heights of monosome peaks as readouts (Supplementary Figure S1). We reasoned that the optimal RNase concentration should be the concentration that converts polysomes to monosomes without substantial ribosomal disassembly and thereby maximizes the RPF yield. Following this rationale, we determined 100 ng RNase A + 60 U RNase T1/*A*_260_ at 25°C for 30 min as the optimal digestion condition (Supplementary Figure S1).

**Figure 1.**
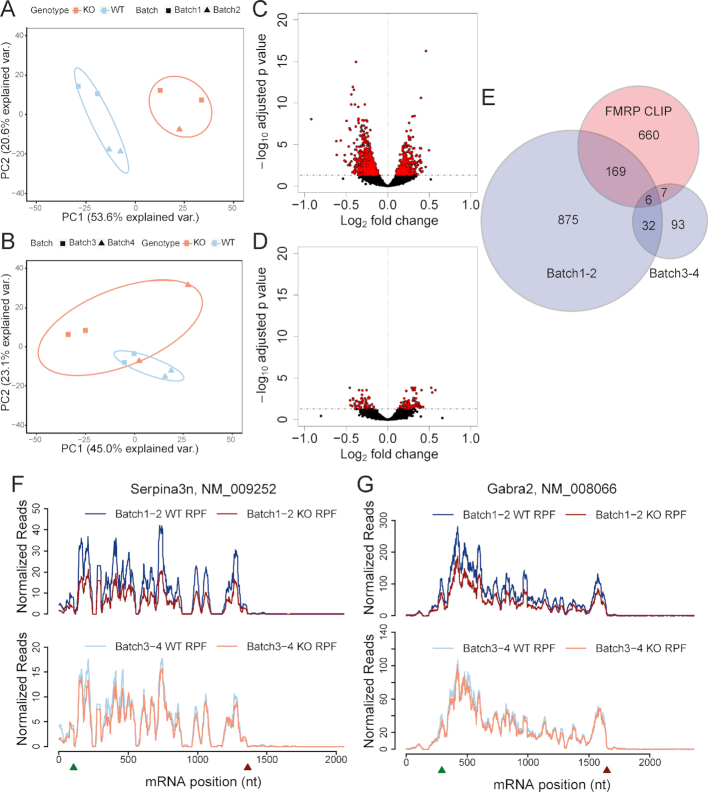
Sample batch effects dramatically compromise the power of ribosome profiling. (**A**) Ribosome protected footprints (RPFs) from batch1–2 samples were mapped to Refseq mouse coding sequence (CDS) reference and quantified with RSEM. Counts were regularized log transformed, normalized with DESeq2, and used for principal component analysis (PCA) using the ‘prcomp’ function from the ‘stats’ package. Samples with the same genotypes are labeled with the same color and circled to show the genotype separation. Batches are labelled with different shapes. PCA analysis shows that the major (PC1) variance (var.) of ribosome profiling data from batch1–2 samples was derived from genotypes. (**B**) PCA analysis shows that the major (PC1) variance (var.) of ribosome profiling data from batch3–4 samples was derived from experimental batches. (**C**) Counts of RPFs mapped to CDS from batch1–2 were batch-corrected with the ‘Combat’ function from ‘sva’ package and used for the differential gene expression analysis with DESeq2. Volcano plot of batch1–2 shows the log_2_ fold changes (KO/WT) of RPF abundance with –log_10_ adjusted *P* value. Differentially expressed genes (DEGs) with adjusted *P* value less than 0.05 are colored red. (**D**) Volcano plot of batch3–4 shows the log2 fold changes (KO/WT) of RPF abundance with –log_10_ adjusted *P* value. DEGs with adjusted *P* value less than 0.05 are colored red. (**E**) Overlap between the top FMRP CLIP genes (32) and DEGs identified with batch1–2 samples (red points in C, *P* = 2.3e–43, hypergeometric test) or batch3–4 samples (red points in D, *P* = 0.03, hypergeometric test). (**F**) RPF distributions on the serine peptidase inhibitor, clade A, member 3N (*Serpina3n*) gene. RPF number at each mRNA nucleotide position was calculated with the ‘plastid’ package, normalized to the library size, averaged across all replicates of batch1–2 (top panel) or batch3–4 (bottom panel), and plotted along the mRNA nucleotide positions with green and red triangles for annotated start and stop codons respectively. For visualization purposes, the curves were smoothed within a 30nt window. (**G**) RPF distributions on the gamma-aminobutyric acid (GABA) A receptor, subunit alpha 2 (*Gabra2*) gene.

We then applied this protocol to investigating the translational dysregulation in fragile X syndrome. This protocol worked well for high-input samples, such as whole cortex from one P35 mouse (∼20 *A*_260_) and cultured mouse adult neural stem cells (∼10 *A*_260_) (36). Next, we applied the same digestion condition to low-input samples: mouse hippocampal slices (∼3 *A*_260_). We first processed two batches of samples, each containing 1–2 replicates of pooled hippocampal slices from wild type (WT) and *Fmr1* KO littermates (6–8 mice/replicate). The principal component analysis (PCA) revealed that ∼60% variances of RPF abundance could be explained by the genotype differences while ∼20% might be caused by batch effects (Figure 1A). However, when we processed a third and fourth batches six months later, ∼50% variances of RPF abundance were from batch effects with only ∼20% variances from genotype differences (Figure 1B). As a result, we detected 2049 differential expressed genes (DEGs) from batch1–2, many more than 250 DEGs from batch 3–4 (Figure 1C and D). Noticeably, the magnitudes of changes were quite modest (<50%) for this comparison, emphasizing the importance of minimizing technical bias to detect low levels of biological signals. To estimate potential false negatives, we compared the overlap between the DEGs found by ribosome profiling and mRNAs directly bound by FMRP uncovered by cross-linking immunoprecipitation (CLIP) (32). DEGs detected with batch1–2 overlapped significantly (*P* = 2.3e–43, hypergeometric test) with FMRP CLIP genes, suggesting that changes of many DEGs were the direct result of the loss of FMRP. In addition, DEGs detected with batch1–2 revealed a decrease of expression for many cell adhesion and synaptic molecules based on Gene Ontology (GO) analysis, which is consistent with the dysregulated neural functions in *Fmr1* KO mice (Supplementary Figure S2). However, significance of the overlap between batch3–4 DEGs and FMRP CLIP genes was only marginal (*P* = 0.03, hypergeometric test), implying a potentially high false negative rate. A number of specific mRNAs such as serine (or cysteine) peptidase inhibitor, clade A, member 3N (*Serpina3n*) and gamma-aminobutyric acid (GABA) A receptor, subunit alpha 2 (*Gabra2*) that were the most differentially expressed in batch1–2 have been previously reported to be dysregulated in *Fmr1* KO brain (37) (Figure 1F and G). Decreased expression of the GABAA receptor was reported in FXS and many drugs that modulate the GABAergic system have already been tested in animal models and clinical trials (38,39). However, both *Serpina3n* and *Gabra2* showed no difference in batch3–4. This again strongly indicates that sample batch effects dramatically compromise the power of ribosome profiling.

### RPF GC content is correlated with sample batch effect

**Figure 2.**
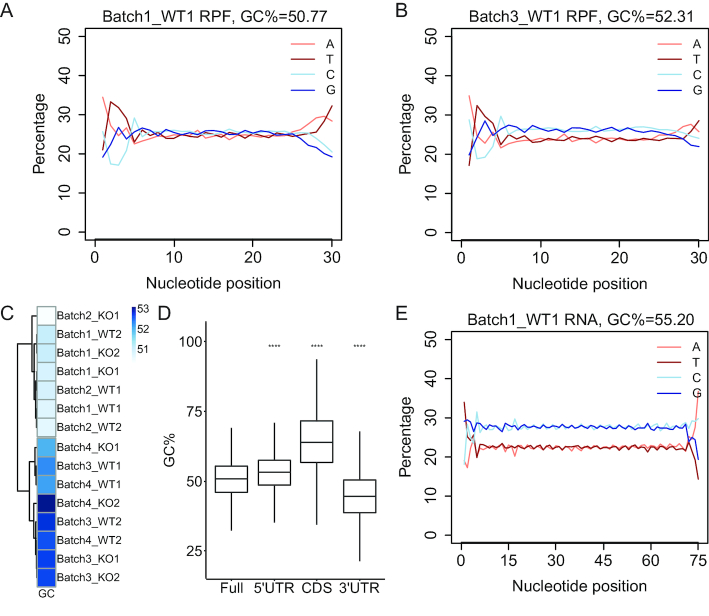
RPF GC content is correlated with sample batch effect. (**A**) Sequences of RPFs mapped to CDS were extracted from bam files with bedtools and samtools. Nucleotide composition at each position of RPFs for a representative sample from batch1 in Figure 1A was calculated and plotted. The mean GC percentage within the 10–20nt window was calculated and shown on the top. (**B**) Nucleotide composition at each position of RPFs mapped to CDS for a representative sample from batch3 in Figure 1B. (**C**) GC contents of RPFs from all the batch1-4 samples were calculated and plotted as a heatmap showing the correlation with the sample batches. Darker blue represents higher GC content. (**D**) The most abundant mRNA isoform in Batch1 WT1 sample estimated by RSEM was selected as the representative transcript for each gene across the Refseq transcriptome. GC contents of sequences for different mRNA regions in the curated and nonredundant transcriptome were calculated and plotted to visualize the medians. The lower and upper hinges correspond to the first and third quartiles. The whiskers extend from the hinges to the largest and smallest values no further than 1.5 fold of inter-quartile range. Outliers are not shown. Using full length of mRNAs as the reference, all pair-wise comparisons are statistically significant (***P < 0.001, Wilcoxon rank sum test). (**E**) Nucleotide composition at each position of RNA-seq reads mapped to CDS for the same sample in (D). The mean GC percentage within the 10–65nt window was calculated and shown on the top.

To identify the potential source(s) of batch effects, we re-analyzed various aspects of our ribosome profiling data. One major concern was the cutting preferences of RNases. RNase A cuts after C and U residues while RNase T1 cuts after G residues, thus a cocktail of RNase A and T1 cannot cut after A residues. To evaluate the potential bias introduced by the cutting preferences, we calculated the base composition at each nucleotide position of RPFs mapped to CDS. As expected, the 5′ ends of RPFs were biased to A (Figure 2A and B). Surprisingly, we found that the batch3–4 RPFs with strong batch effects were slightly GC rich across the entire length of the footprint read, which could not readily be explained by the specificities of RNases (Figure 2B). We also observed a correlation between higher RPF GC content and strong batch effect (Figure 2C, Supplementary Table S1). Because RPFs are derived from mRNA CDS, we calculated the base compositions for the mouse reference transcriptome (Figure 2D) and RNA-seq reads mapped to CDS (Figure 2E). We found that GC content of mRNA CDS was >50%, which is counterintuitive, because GC contents are expected to be similar for both mRNA reads mapped to CDS and RPFs.

### RPF GC content is RNase-species independent

To determine the experimental step in which the GC-content correlated batch effects were introduced, we compared a series of factors including fresh vs frozen samples, lysis buffer with or without detergent, lysate quantification with Nanodrop or Qubit assays, RNA extraction with TRIzol and isopropanol precipitation versus Zymo column (40), monosome fraction sample with or without sucrose, and the M. Moore library preparation protocol (34) compared to the N. Ingolia protocol (41). None of the above caused the GC-content correlated batch effects (data not shown). The most prominent remaining variable was the RNase digestion step.

**Figure 3.**
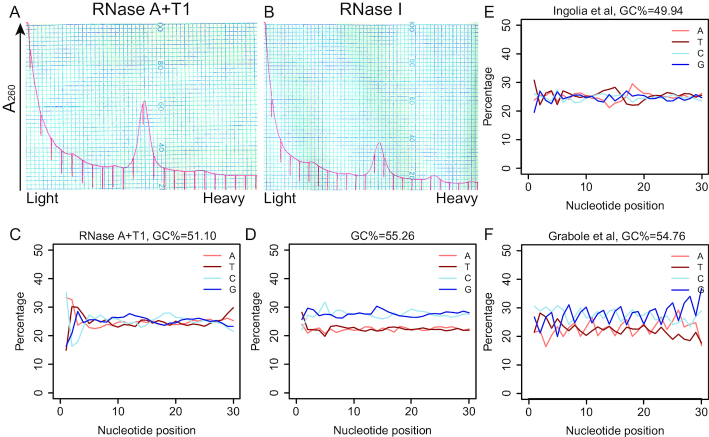
RPF GC content is RNase-species independent. (**A**) 3.8 A_260_ homogenate from hippocampi of one P35 male mouse was digested with 100ng RNase A (Sigma, # R4875) + 60U RNase T1 (Thermo Fisher Scientific, #EN0542)/*A*_260_, at 25°C for 30min and applied to a 10–50% (w/v) sucrose gradient. (**B**) 3.8 *A*_260_ homogenate from hippocampi of one P35 mouse was digested with 5U RNase I (Ambion, #AM2294)/*A*_260_, at 25°C for 45min and applied to a 10–50% (w/v) sucrose gradient. (**C**) Nucleotide composition at each position of RPFs mapped to CDS from ribosomes in (A). (**D**) Nucleotide composition at each position of RPFs mapped to CDS from ribosomes in (B). (**E**) Nucleotide composition at each position of RPFs mapped to CDS from mouse embryonic stem cells (mESCs) (data from Ingolia *et al.*) (16). A 600 μl aliquot of lysate was treated with 15 μl RNase I at 100 U/μl for 45 min at 25°C. (**F**) Nucleotide composition at each position of RPFs mapped to CDS from human embryonic stem cell (hESC)-derived neurons (data from Grabole *et al.*) (42). 5 U TruSeq Ribo Profile Nuclease/*A*_260_ at 25°C for 45 min.

Most ribosome profiling studies utilize RNase I and report no correlation between batch effects and GC content. Thus, we were concerned that the issue of GC content/ batch effects might be related to the RNase A+T1 cocktail. To assess this possibility, we performed ribosome profiling on a WT mouse hippocampal sample treated with either RNase A+T1 or RNase I. Because RNase I cuts after all four bases without preference, the stronger activity of this enzyme led to partial disassembly of monosomes (Figure 3A and B). For this particular experiment, RPFs with RNase A+T1 were not GC-rich (Figure 3C), but RNase I led to RPFs with higher GC content (Figure 3D). To exclude the possibility that the high GC content was introduced from our library preparation protocols, we calculated the GC contents of RNase I-generated RPFs from multiple published studies. Most RPFs from proliferative cells were not GC rich, for example, the RPFs from mouse ES cells generated by Ingolia *et al.* (Figure 3E) (16). However, RPFs from neuronal cultures showed substantially higher GC content in several studies, for example RPFs from human ES cell-derived neurons by Grabole *et al.* (Figure 3F) (42). Therefore, RPF GC content is independent of the particular RNase used and appears to mainly occur in samples with low input amount or low translational activity.

### RPF GC content and length depend on the RNase digestion protocol

**Figure 4.**
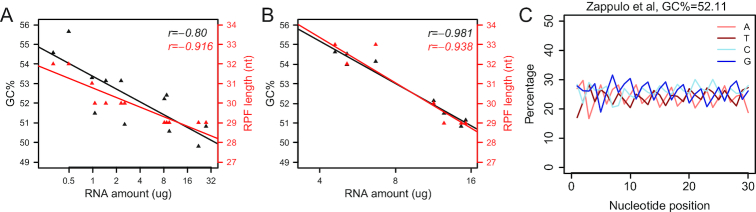
RPF GC content and length depend on the RNase digestion protocol. (**A**) Lysates from human iPSC neuron samples spanning a wide range of amounts were digested with 100 ng RNase A + 60U RNase T1/*A*_260_ at 25°C for 30 min. Monosomal RNA was extracted from monosomal fractions of sucrose gradients and quantified with Nanodrop. GC contents were calculated as in Figure 2A and the peaks of length distributions of RPFs mapped to CDS were also determined. Scatter plots with Pearson correlation coefficients show the negative correlation between 80S monosomal RNA amounts (log_2_ scale) and the GC contents (black) or RPF lengths (red). (**B**) Lysates from human iPSC samples were digested with 20 ng RNase A + 12 U RNase T1/*A*_260_ at 25°C for 30 min. Scatter plots with Pearson correlation coefficients show the negative correlation between 80S monosomal RNA amounts (log_2_ scale) and the GC contents (black) or RPF lengths (red). (**C**) Nucleotide composition at each position of RPFs mapped to CDS from mESC-derived neurons with an alternative protocol of RNase digestion (data from Zappulo *et al.*) (43). 70 U RNase I at 25°C for 40 min.

To examine whether there is indeed a correlation between RPF GC content and sample amount, we analyzed ribosome profiling data from a batch of human iPSC-neuron samples with a wide range of lysate amounts. Different iPSC lines showed distinct capabilities of differentiation into various neural lineages, including progenitors, glia, or post-mitotic neurons. Thus, samples of iPSC neuron cultures had vastly different translational activities and input amounts. Those samples were processed at the same time as one batch. A clear and strong negative correlation was observed between the GC content and 80S monosomal RNA amount, which is an indirect estimate of lysate amount (Figure 4A). Initially, we reasoned that the low-input samples might be sensitive to over-digestion by RNase, whereby retained ribosomes would preferentially contain GC-enriched footprints. To test this hypothesis, we processed another batch of samples with 5-fold diluted RNase concentrations. However, the negative correlation between GC content and lysate amount remained (Figure 4B). Furthermore, Zappulo *et al.* performed ribosome profiling on neurite-localized ribosomes (43), an extremely low abundance sample, leaving those investigators to omit the ribosome isolation step. Even with this modified method, the yield of monosomal RNA (400 ng) was still much lower than our samples (Figure 4A and B). However, their RPFs did not have a high GC content (Figure 4C). Collectively, these results suggest that high RPF GC content is not necessarily caused by low sample amount.

When we carefully compared the experimental methods used by Grabole *et al.* (Figure 3F) and Zappulo *et al.* (Figure 4C), we recognized that the RNase amounts used for digestion were dramatically different. Grabole *et al.* followed the manual of TruSeq Ribo Profile kit and digested the lysates with 5 U TruSeq Ribo Profile Nuclease/*A*_260_ at 25°C for 45 min. In contrast, Zappulo *et al.* did not adjust the RNase I amount based on the *A*_260_ but instead digested the lysates with the fixed amount of 70 U RNase I at 25°C for 40 min. Considering the extremely low amounts of ribosomes in the neurites, the RNase to RNA ratio in Zappulo *et al.* was much higher than that in Grabole *et al.* This seemed counterintuitive: low-input samples digested with high amounts of RNase generated RPFs without GC bias and presumably with smaller batch effects. Thus, we reasoned that RPF GC content depends on the RNase digestion protocol.

### The GC-content correlated batch effects are caused by incomplete RNase digestion

In addition to GC content, we also found a negative correlation between RPF lengths and sample amounts (Figure 4A and B). Low-input samples had longer RPFs, an indication of incomplete RNase digestion. Therefore, we hypothesized that the GC-content correlated batch effects are caused by the incomplete RNase digestion. The problem arises from the adjustment of RNase amount based on the sample amount, which leads to varied RNase concentrations in the digestion reaction. The input mRNA is GC-rich (∼60%) for CDS in the lysate. For low-input samples, the adjusted RNase concentration is low and the batch effects reflect the extents of limited RNase digestion (various GC contents and RPF lengths). For large input samples, the RNase concentration is sufficiently high, so most samples are completely digested (∼50% GC and ∼28nt RPF length), resulting in much smaller batch effects.

To directly test this hypothesis, we performed a 5-fold serial titration experiment of RNase A+T1 with low-input hippocampal lysates from a P35 WT mouse. 100 ng RNase A+ 60 U T1 per *A*_260_ was the condition we first established with high-input mouse cortical samples (Supplementary Figure S1). For one experiment (not shown), we used a new aliquot of RNase A (Sigma, #R4875, dissolved in water) that did not go through freeze-thaw cycles and followed the same formula. The digestion resulted in a complete disassembly of ribosomes, so we realized that the old RNase A activity had declined through freeze-thaw cycles. For the titration experiments, we decided to switch to a better quality controlled and more stable RNase A (Ambion, #AM2270). This brand of enzyme is stored in a buffer with 50% glycerol that hopefully could reduce the rate of activity decline.

**Figure 5.**
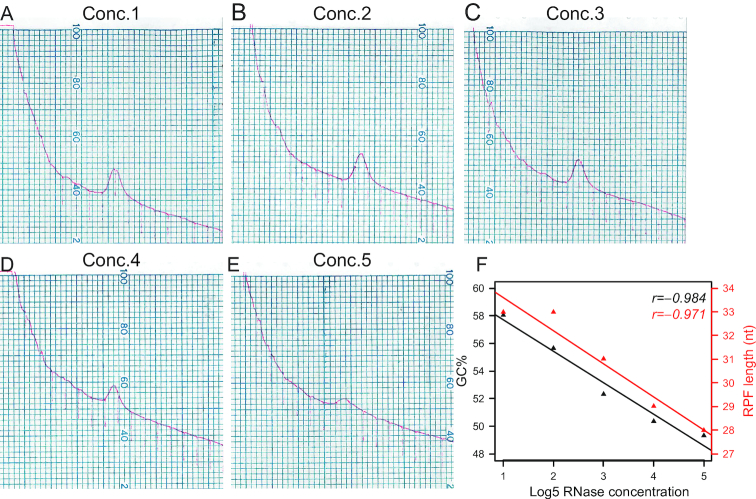
The GC-content correlated batch effects are caused by incomplete RNase digestion. (**A**) Hippocampi from one P35 WT mouse were homogenized and the homogenate was aliquoted for the titration experiment. 0.5 unit *A*_260_ homogenate containing 2 μg RNA (measured with Qubit HS RNA kit) in 0.3 ml volume was used for digestion at each RNase concentration. Digested homogenates were separated on 10–50% (w/v) sucrose gradients. Profile of hippocampal ribosomes after the digestion at the lowest concentration1 [Conc.1, 4.8ng RNase A (Ambion, #AM2270) + 0.6 U RNase T1 (Thermo Fisher Scientific, #EN0542)/μg RNA × 2 μg RNA in 0.3 ml at 25°C for 30 min] and sucrose gradient fractionation. (**B**) Profile of hippocampal ribosomes after the digestion at the concentration2 (Conc.2, 24 ng RNase A + 3U RNase T1/μg RNA × 2 μg RNA in 0.3 ml at 25°C for 30 min) and sucrose gradient fractionation. (**C**) Profile of hippocampal ribosomes after the digestion at the concentration3 (Conc.3, 120 ng RNase A + 15 U RNase T1/μg RNA × 2 μg RNA in 0.3 ml at 25°C for 30 min) and sucrose gradient fractionation. (**D**) Profile of hippocampal ribosomes after the digestion at the concentration4 (Conc.4, 600 ng RNase A + 75 U RNase T1/μg RNA × 2 μg RNA in 0.3 ml at 25°C for 30 min) and sucrose gradient fractionation. (**E**) Profile of hippocampal ribosomes after the digestion at the highest concentration5 (Conc.5, 3000 ng RNase A + 375 U RNase T1/μg RNA × 2 μg RNA RNA in 0.3 ml at 25°C for 30 min) and sucrose gradient fractionation. (**F**) Scatter plots with Pearson correlation coefficients show the negative correlation between RNase concentrations (log_5_ scale) and the GC contents (black) or RPF lengths (red).

Across a wide-range of RNase concentrations (Conc.1–3), no obvious difference was observed for monosome peak heights in the sucrose gradient profiles, suggesting that this is not a particularly informative readout for the optimal RNase concentration (Figure 5A–C). With higher RNase concentrations, the monosomes were slightly disassembled (Conc.4) and substantially disassembled (Conc.5) (Figure 5D–E). Next, we confirmed that both the GC contents and RPF lengths negatively correlate with the RNase concentrations and only very high RNase concentrations (Conc.4–5) generated RPFs with the optimal GC content (∼50%) and length (28–29nt), as in the batch1–2 samples without strong batch effects (Figure 2). These results directly validate the idea that the GC-content correlated batch effects are caused by the incomplete RNase digestion.

### Optimized RNase digestion generates ribosome profiling data with higher quality and reproducibility

**Figure 6.**
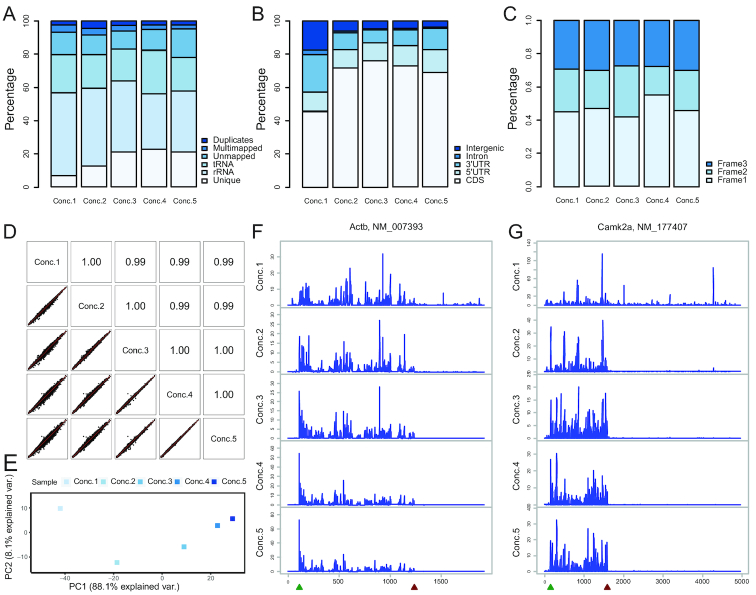
Optimized RNase digestion generates ribosome profiling data with higher quality and reproducibility. (**A**) rRNA and tRNA contaminates were filtered out from RPF reads with Bowtie2 and unmapped reads were next mapped to mm10 with Tophat2. ‘Unmapped’ and ‘Multimapped’ reads were defined based on the Tophat2 outputs. PCR-derived ‘Duplicates’ were identified based on the unique molecule identifier (UMI). The uniquely mapped reads after all the upstream filtering were classified as ‘Unique’ and used for downstream analyses. Stacked bar plots show the percentage of RPFs uniquely mapped to the transcriptome under different RNase concentrations. (**B**) The number of ‘Unique’ reads in (A) mapped to various gene regions were calculated by intersecting bam files with USCS bed annotations by bedtools and samtools. Stacked bar plots show the percentage of ‘Unique’ RPFs in (A) mapped to CDS under different RNase concentrations. (**C**) The P-site offsets and frame preferences of ‘Unique’ RPFs in (A) mapped to CDS were calculated with ‘plastid’ package. The best frame resolution across all the RPF lengths was selected to represent each RNase concentration. Stacked bar plots show the percentage of ‘Unique’ RPFs in (A) mapped to the same frame as the annotated CDS (Frame1) under different RNase concentrations. (**D**) ‘Unique’ reads in (A) were mapped to Refseq mouse CDS reference and quantified with RSEM. Counts of mapped RPFs were regularized log transformed and normalized with DESeq2. Scatter plots and Pearson correlation coefficients show the reproducibility of RPF abundance quantification among various RNase concentrations. (**E**) Principal component analysis (PCA) shows the similarity between Conc.4 and Conc.5 samples. (**F**) Distinct RPF distributions under different RNase concentrations on actin beta (*Actb*) mRNA. RPF number at each mRNA nucleotide position was calculated with the ‘plastid’ package, normalized to the mean read density on CDS, and plotted along the mRNA nucleotide positions with green and red triangles for annotated start and stop codons respectively. (**G**) Distinct RPF distributions under different RNase concentrations on the calcium/calmodulin-dependent protein kinase II alpha (*Camk2a*) mRNA.

We next asked whether the extent of RNase digestion influenced the quality and reproducibility of ribosome profiling. Although monosome peak heights looked comparable, the uniquely mapped reads increased while rRNA/tRNA contaminants decreased with increasing RNase concentrations (Figure 6A). For the lowest Conc.1 concentration, substantial amounts of reads were mapped to regions outside of CDS, suggesting incomplete digestion and false signals not representative of true translation (Figure 6B). In addition, the Conc.4 condition produced RPFs with the best three-nucleotide periodicity, which was considerably compromised with incomplete- (Conc.1–3) or over-digestion (Conc.5) (Figure 6D).

In addition to general quality, we also evaluated reproducibility with increasing RNase concentrations. Although the pair-wise correlations of gene expressions all seemed high, the variances were larger with lower RNase concentrations (Figure 6D). In contrast, almost all genes were clustered tightly along the diagonal line when comparing Conc.4 and 5. PCA analysis consistently revealed that samples Conc.4 and 5 with higher RNase concentrations were clustered together, indicating lower variances introduced by enzyme digestion (Figure 6E). Finally, because local ribosome densities are often used to infer translational pausing, we also examined the impact of RNase digestion on this property. Consistent with the distribution of footprints on various RNA regions shown in Figure 6B, a substantial number of reads were mapped to 3′UTR regions under Conc.1 conditions (Figure 6F and G), which may represent mRNA fragments protected by RNA binding proteins. Importantly, local ribosome densities and peaks varied dramatically among RNase concentrations and became more reproducible under higher RNase concentrations. In summary, optimal RNase digestion is essential to ensure high quality and reproducibility of ribosome profiling data especially for low-input samples where modest changes in ribosome densities are expected.

## DISCUSSION

Ribosome profiling is a valuable tool for studying mRNA translation because of its unprecedented resolution. Dramatic translational changes could be reliably captured by ribosome profiling, for instance in cultured cells under a stress response (15) or upon mTOR pathway manipulation (44). In contrast, the magnitude of translational changes in neural systems are often much more modest. One possible explanation is that chronic loss of function in mouse knockout models leads to an adaptation response by compensatory mechanisms at the transcription, RNA stability, or translational levels. Another possibility is that the cell-type specific changes might be ‘diluted’ by diverse cell types in the brain. Translating ribosome affinity purification (TRAP) is an alternative approach to monitor cell-type specific expression changes (45), but TRAP is generally unable to delineate the number of ribosomes bound to an mRNA, thus further complicating determinations of translational efficiency. Therefore, with currently available technologies, it is imperative to improve the reproducibility of ribosome profiling to capture subtle changes when comparing across genotypes or disease models such as the FXS example (Figure 1).

Mammalian ribosome profiling has been performed using various RNase species with distinct cutting patterns, including RNase I and RNase A+T1 cocktail (22). When the sample amounts are not limited, RNase I has the major advantage of high frame resolution. However, we noticed that RNase I caused a substantial disassembly of monosomes for mouse cortical samples (Supplementary Figure S1) as previously reported for other types of mammalian samples (35). For low input samples from brain tissue or neural cultures, RNase I results in very low yields of monosomal RNA, making the library preparation extremely difficult (the minimum input for the RiboZero kit is 1μg) and prone to technical biases. The RNase A+T1 cocktail maintains the integrity of monosomes better thus leads to higher yields of monosomal RNA but at the cost of lower frame resolution due to the base cutting preferences (Supplementary Figure S1 and Figure 6C). mRNA-level resolution is sufficient for calculating translational efficiencies, so we chose the RNase A+T1 cocktail to maximize the yield of monosomal RNA for the input of library preparation.

Our data revealed that inconsistent RNase digestion was a major contributor to poor reproducibility in ribosome profiling, especially, of low-input samples with small changes in footprint densities. Theoretically, the most consistent results can be obtained by digesting the same amounts of lysates with the same amounts of RNase at a fixed volume, temperature and time (46). However, descriptions of how to optimize the RNase concentration for different types of samples are lacking. In practice, different samples can have a wide range of RNA amounts, making it unfeasible to adjust the digestion amounts for low-input samples. Instead, many ribosome profiling studies adjust the amount of RNase in the digestion based on the amounts of nucleic acids (usually A_260_ units) in the lysates and scale down linearly for low-input samples. However, we find that this step, while seemingly appropriate, introduces unexpected inconsistences. Therefore, we propose a better practice of RNase digestion optimization. In designing new ribosome profiling experiments, an RNase titration experiment covering a wide-range of concentrations should be performed on representative test samples that mimic the amounts of real samples. Because sucrose gradient profiles are not reliable readouts for determining the optimal RNase concentration (Figure 5A–E), libraries should ideally be prepared, sequenced at a relatively low depth and assessed for specific quality control metrics, primarily the GC content and length of RPF (Figure 5 and 6). Ideally, the same RNA to RNase ratio in a fixed digestion volume should be maintained for all samples to obtain most consistent results. However, to maximize the RNA yield, it is often difficult to adjust low-input samples to the same amount. In this case, we suggest to use a relatively high concentration of RNase to achieve more complete digestion and consequently more reproducible results (Figure 6D and E). The initial RNase concentration that we used for the FXS hippocampi batch1–2 might be marginally enough. However, the RNase activity was probably reduced through freeze-thaw cycles over the six month period and led to incomplete digestion as well as strong batch effects for batch3–4 (Figure 1). Batch3–4 samples were collected freshly and processed within one week. In addition, iPSC samples (Figure 4A and B) were collected within one week but still showed variable GC contents. These results argue against the sample degradation as a major contributor of GC content.

One puzzling result is the inconsistency between the GC contents of input mRNA CDS and RPFs without batch effects. If RPFs are samplings of mRNA CDS, they should have a similar GC content. However, the mRNA CDS was slightly GC-rich (∼60%), while the RPFs with little batch effect had ∼50% GC. Those RPFs exhibited higher reproducibility statistically, but it is unclear whether they reflect the true ribosome occupancies *in vivo*. One explanation is that complete RNase digestion only retains a subset of RPFs that are most resistant to RNase and most reproducible across conditions. Another interesting observation is that different species of RNases seem to have dramatically different kinetics to achieve complete digestion. RNase I cut after all four bases as compared to only U,C,G for RNase A+T1, thus RNase I should have stronger activity. Indeed, RNase I caused more monosome disassembly than RNase A+T1 shown in sucrose gradient profiles (Figure 3A and B), but the corresponding RNase I digestion was not complete for ribosome profiling as indicated by high RPF GC content and long RPF length (Figure 3D). This result emphasizes once again that the integrity of monosome peak is not a reliable readout of ribosome profiling quality. Indeed, generation of high quality ribosome profiling data required much more RNase I, which almost completely dissembled the monosome peak (22). The underlying mechanism for this kinetic difference is unclear, indicating that each RNase must be characterized empirically.

Several recent studies compared the impact of different RNases on ribosome profiling data (22,47) and found that different RNases generated ribosome footprints with distinct size distributions. Combined with our results, the differences were not only caused by distinct enzymatic specificities but probably also the extent of digestion. Fragment length metric (FLOSS) analysis has been used to identify true ribosome footprints bioinformatically (48), which could be seriously compromised with various levels of RNase digestion. Gerashchenko *et al.* (22) also showed that different RNases yielded comparable estimations of gene expression when ribosome integrity is not compromised. Similarly, our data describing different concentrations of the same RNase also exhibited high correlation of RPF abundance (Figure 6D). However, the ‘completeness’ of RNase digestion did influence the identification of differentially expressed genes with subtle changes significantly (Figure 1). Finally, Gerashchenko *et al* showed that ribosome coverage patterns of individual transcripts had little in common among the RNases. In addition to the distinct cutting preferences of RNases, various extents of digestion of the same RNase also led to dramatically different ribosome coverage patterns (Figure 6F and G). Therefore, inconsistent RNase digestion might partially explain the substantial variation of local ribosome densities across laboratories (29). This observation also implies that certain peaks of ribosome footprints might not represent true elongation pausing sites, which could be a major cause of inconsistent results on translational pausing and codon usage (27,28).

In summary, we present a comprehensive analysis of the impact of RNase digestion on the quality and reproducibility of ribosome profiling data especially for low-input samples with small changes in footprint densities. We establish a complete workflow with quality control metrics for the optimization of RNase digestion, which is often underappreciated. Our data provide guidance for a better interpretation of ribosome density data and pave the way for the application of ribosome profiling to investigating neurological diseases.

## DATA AVAILABILITY

The raw sequencing data reported in this paper have been deposited in GEO under accession number GSE116233. Core scripts for the data analysis and plotting have been deposited to GitHub (https://github.com/botaoliuphd/rnase_manuscript).

## Supplementary Material

Supplementary Data
