# Supplementary material for: Optimization of ribosome profiling using low-input brain tissue from fragile X syndrome model mice
Source: Nucleic Acids Res. 2018 Dec 24;47(5):e25. doi: 10.1093/nar/gky1292 (PMC6411937; doi:10.1093/nar/gky1292)
Supplement: Supplementary Data [file gky1292_supplemental_files.pdf]

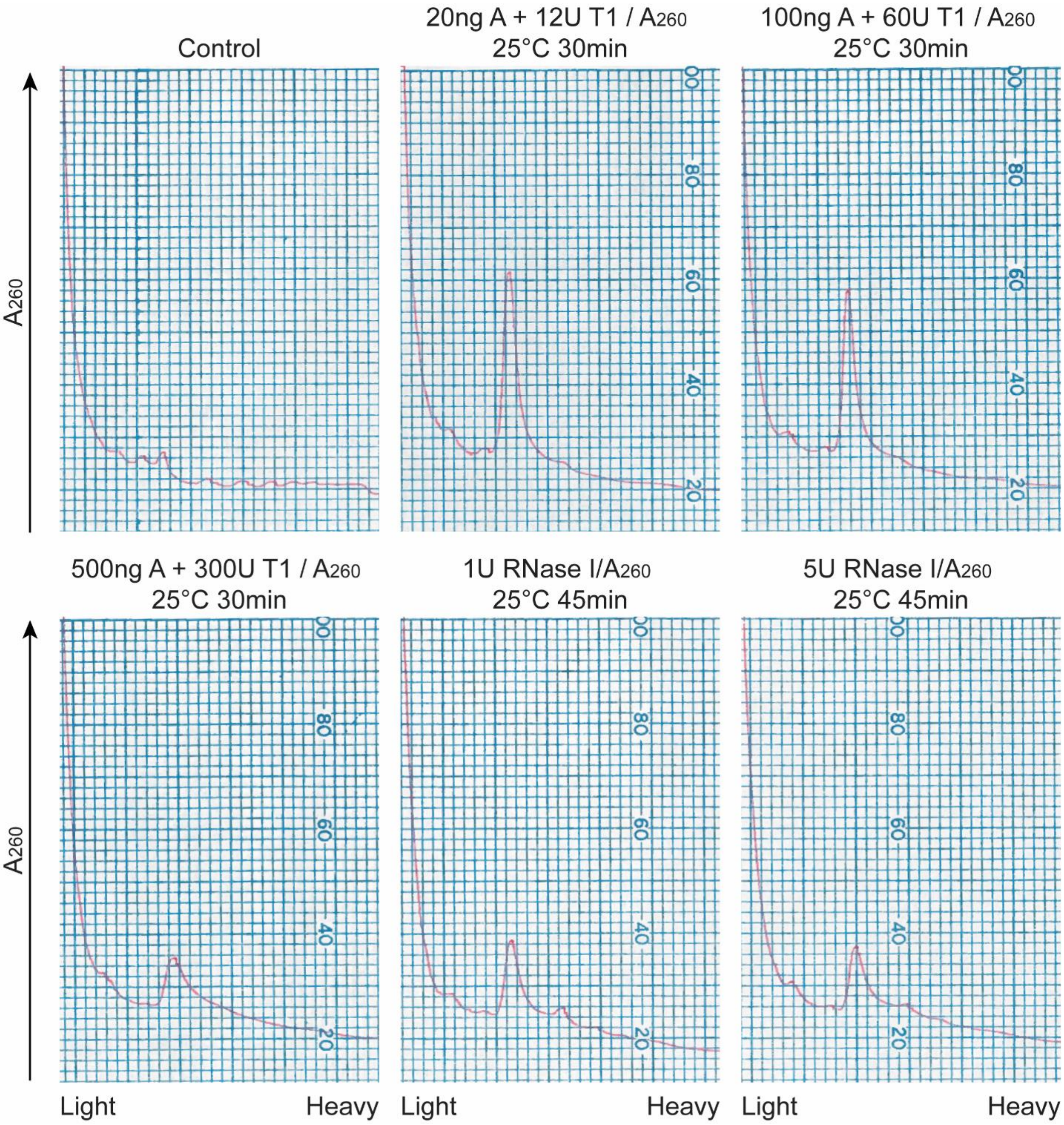

Figure S1. Comparison of different RNase digestion conditions with mouse cortical samples

10 A_{260} units of cortical homogenates from P35 WT mice were digested with RNase under indicated conditions and applied to 10-50% (w/v) sucrose gradients. After the ultracentrifugation, gradients were fractionated through a fractionation system that continually monitored A_{260} absorbance at the same 2.0 sensitivity.

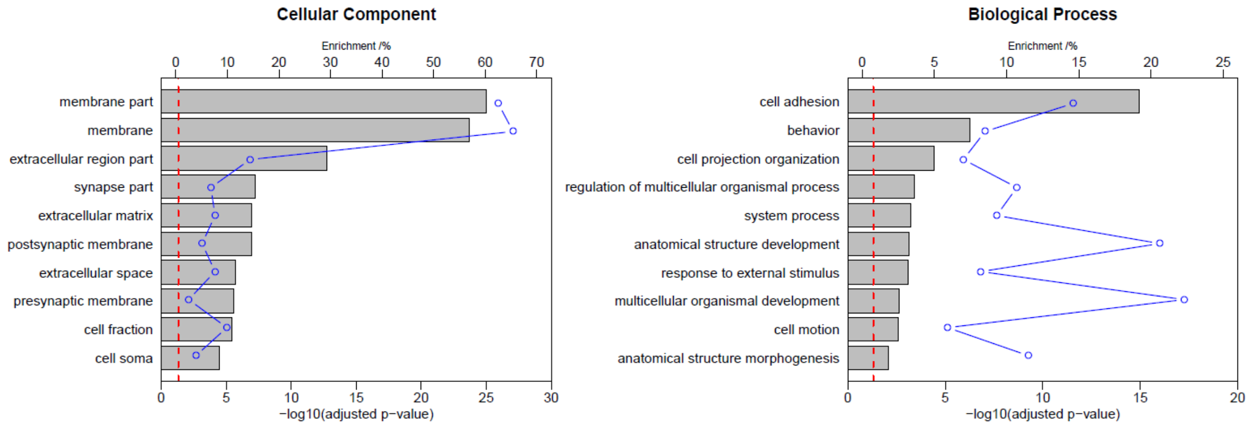

Figure S2. Gene Ontology (GO) term analyses for DEGs with decreased RPFs in *Fmr1* KO mice from batch1-2 samples

The enrichment percentages are plotted as connected blue lines on the top axis. The significance (-$\log_{10}$ adjusted p-value) of enrichment is represented as bar plots on the bottom axis; the red dash line denotes a 0.05 cut-off.

Table S1. Summary of samples used in this paper.

GEO Accession	Name	A260 units	Digestion Conditions	Source	RNA yield (µg)	GC %	Length (nt)
GSM3214384	rnase1	0.5 (2µg RNA)	4.8ng RNase A + 0.6U RNase T1/µg RNA × 2µg RNA in 0.3ml at 25°C for 30min	RPF	0.61	58.07	33
GSM3214385	rnase2	0.5 (2µg RNA)	24ng RNase A + 3U RNase T1/µg RNA × 2µg RNA in 0.3ml at 25°C for 30min	RPF	0.69	55.67	33
GSM3214386	rnase3	0.5 (2µg RNA)	120ng RNase A + 15U RNase T1/µg RNA × 2µg RNA in 0.3ml at 25°C for 30min	RPF	0.66	52.31	31
GSM3214387	rnase4	0.5 (2µg RNA)	600ng RNase A + 75U RNase T1/µg RNA × 2µg RNA in 0.3ml at 25°C for 30min	RPF	0.57	50.35	29
GSM3214388	rnase5	0.5 (2µg RNA)	3000ng RNase A + 375U RNase T1/µg RNA × 2µg RNA RNA in 0.3ml at 25°C for 30min	RPF	0.42	49.32	28
GSM3396959	qc_sample_wt_rpf_batch1_rep1	2.10	100ng RNase A + 60U RNase T1/ A260, at 25°C for 30min	RPF	2.43	50.77	29
GSM3396960	qc_sample_wt_rpf_batch1_rep2	1.91	100ng RNase A + 60U RNase T1/ A260, at 25°C for 30min	RPF	3.11	51.11	29
GSM3396961	qc_sample_wt_rpf_batch2_rep1	2.62	100ng RNase A + 60U RNase T1/ A260, at 25°C for 30min	RPF	4.33	50.89	29
GSM3396962	qc_sample_wt_rpf_batch2_rep2	2.10	100ng RNase A + 60U RNase T1/ A260, at 25°C for 30min	RPF	4.43	50.72	30
GSM3396963	qc_sample_wt_rpf_batch3_rep1	1.57	100ng RNase A + 60U RNase T1/ A260, at 25°C for 30min	RPF	4.70	52.31	30
GSM3396964	qc_sample_wt_rpf_batch3_rep2	1.45	100ng RNase A + 60U RNase T1/ A260, at 25°C for 30min	RPF	4.95	52.83	30
GSM3396965	qc_sample_wt_rpf_batch4_rep1	2.19	100ng RNase A + 60U RNase T1/ A260, at 25°C for 30min	RPF	5.82	52.18	29
GSM3396966	qc_sample_wt_rpf_batch4_rep2	2.01	100ng RNase A + 60U RNase T1/ A260, at 25°C for 30min	RPF	5.72	52.65	30
GSM3396967	qc_sample_ko_rpf_batch1_rep1	2.25	100ng RNase A + 60U RNase T1/ A260, at 25°C for 30min	RPF	5.72	50.88	29
GSM3396968	qc_sample_ko_rpf_batch1_rep2	2.03	100ng RNase A + 60U RNase T1/ A260, at 25°C for 30min	RPF	4.69	51.08	29
GSM3396969	qc_sample_ko_rpf_batch2_rep1	2.18	100ng RNase A + 60U RNase T1/ A260, at 25°C for 30min	RPF	3.14	50.38	29
GSM3396970	qc_sample_ko_rpf_batch3_rep1	1.24	100ng RNase A + 60U RNase T1/ A260, at 25°C for 30min	RPF	4.93	52.75	30
GSM3396971	qc_sample_ko_rpf_batch3_rep2	1.54	100ng RNase A + 60U RNase T1/ A260, at 25°C for 30min	RPF	6.74	52.71	30
GSM3396972	qc_sample_ko_rpf_batch4_rep1	1.93	100ng RNase A + 60U RNase T1/ A260, at 25°C for 30min	RPF	5.75	52.03	29
GSM3396973	qc_sample_ko_rpf_batch4_rep2	1.87	100ng RNase A + 60U RNase T1/ A260, at 25°C for 30min	RPF	3.25	53.12	29

GSM3396974	qc_sample_wt_rna_batch1_rep1	0.70	NA	mRNA	5.08	55.20	NA
GSM3396975	qc_sample_wt_rna_batch1_rep2	0.63	NA	mRNA	4.44	54.74	NA
GSM3396976	qc_sample_wt_rna_batch2_rep1	0.87	NA	mRNA	5.75	55.31	NA
GSM3396977	qc_sample_wt_rna_batch2_rep2	0.70	NA	mRNA	4.87	55.56	NA
GSM3396978	qc_sample_wt_rna_batch3_rep1	0.37	NA	mRNA	5.38	53.88	NA
GSM3396979	qc_sample_wt_rna_batch3_rep2	0.34	NA	mRNA	4.97	54.54	NA
GSM3396980	qc_sample_wt_rna_batch4_rep2	0.47	NA	mRNA	5.32	54.80	NA
GSM3396981	qc_sample_ko_rna_batch1_rep1	0.75	NA	mRNA	6.41	54.23	NA
GSM3396982	qc_sample_ko_rna_batch1_rep2	0.68	NA	mRNA	5.13	54.32	NA
GSM3396983	qc_sample_ko_rna_batch2_rep1	0.72	NA	mRNA	4.22	56.13	NA
GSM3396984	qc_sample_ko_rna_batch3_rep1	0.34	NA	mRNA	4.35	54.54	NA
GSM3396985	qc_sample_ko_rna_batch3_rep2	0.36	NA	mRNA	5.40	54.14	NA
GSM3396986	qc_sample_ko_rna_batch4_rep1	0.45	NA	mRNA	4.59	54.46	NA
GSM3396987	qc_sample_ko_rna_batch4_rep2	0.44	NA	mRNA	4.20	54.59	NA
GSM3396988	qc_sample_hip_at_rpf	3.80	100ng RNase A + 60U RNase T1/ A260, at 25°C for 30min	RPF	8.80	51.10	NA
GSM3396989	qc_sample_hip_i_rpf	3.80	5U RNase I / A260, at 25°C for 45min	RPF	6.90	55.26	NA
GSM3396990	qc_sample_ipsc1_low_rnase	0.74	20ng RNase A + 12U RNase T1/ A260 at 25°C for 30min	RPF	6.67	54.14	33
GSM3396991	qc_sample_ipsc2_low_rnase	0.60	20ng RNase A + 12U RNase T1/ A260 at 25°C for 30min	RPF	4.59	54.61	33
GSM3396992	qc_sample_ipsc3_low_rnase	0.60	20ng RNase A + 12U RNase T1/ A260 at 25°C for 30min	RPF	5.13	54.51	32
GSM3396993	qc_sample_ipsc4_low_rnase	0.57	20ng RNase A + 12U RNase T1/ A260 at 25°C for 30min	RPF	5.12	53.99	32
GSM3396994	qc_sample_ipsc5_low_rnase	2.07	20ng RNase A + 12U RNase T1/ A260 at 25°C for 30min	RPF	15.25	51.14	29
GSM3396995	qc_sample_ipsc6_low_rnase	1.61	20ng RNase A + 12U RNase T1/ A260 at 25°C for 30min	RPF	12.50	51.50	29
GSM3396996	qc_sample_ipsc7_low_rnase	1.84	20ng RNase A + 12U RNase T1/ A260 at 25°C for 30min	RPF	14.63	50.82	29
GSM3396997	qc_sample_ipsc8_low_rnase	1.52	20ng RNase A + 12U RNase T1/ A260 at 25°C for 30min	RPF	11.37	52.13	30
GSM3396998	qc_sample_ipsc_neuron1	0.76	100ng RNase A + 60U RNase T1/ A260, at 25°C for 30min	RPF	1.48	53.15	30

GSM3396999	qc_sample_ipsc_neuron2	3.09	100ng RNase A + 60U RNase T1/ A260, at 25°C for 30min	RPF	8.15	52.21	29
GSM3397000	qc_sample_ipsc_neuron3	2.78	100ng RNase A + 60U RNase T1/ A260, at 25°C for 30min	RPF	9.42	50.55	29
GSM3397001	qc_sample_ipsc_neuron4	0.54	100ng RNase A + 60U RNase T1/ A260, at 25°C for 30min	RPF	0.99	53.30	31
GSM3397002	qc_sample_ipsc_neuron5	1.22	100ng RNase A + 60U RNase T1/ A260, at 25°C for 30min	RPF	2.56	50.91	30
GSM3397003	qc_sample_ipsc_neuron6	1.28	100ng RNase A + 60U RNase T1/ A260, at 25°C for 30min	RPF	2.31	53.15	30
GSM3397004	qc_sample_ipsc_neuron7	0.20	100ng RNase A + 60U RNase T1/ A260, at 25°C for 30min	RPF	0.50	55.64	32
GSM3397005	qc_sample_ipsc_neuron8	7.59	100ng RNase A + 60U RNase T1/ A260, at 25°C for 30min	RPF	22.13	49.81	29
GSM3397006	qc_sample_ipsc_neuron9	9.64	100ng RNase A + 60U RNase T1/ A260, at 25°C for 30min	RPF	27.59	50.82	29
GSM3397007	qc_sample_ipsc_neuron10	0.13	100ng RNase A + 60U RNase T1/ A260, at 25°C for 30min	RPF	0.32	54.57	32
GSM3397008	qc_sample_ipsc_neuron11	0.94	100ng RNase A + 60U RNase T1/ A260, at 25°C for 30min	RPF	1.07	51.49	30
GSM3397009	qc_sample_ipsc_neuron12	2.89	100ng RNase A + 60U RNase T1/ A260, at 25°C for 30min	RPF	8.73	52.40	29

Table S2. Oligonucleotides used in this paper.

**Description**	**Sequence (5’-3’)**
Marker: 26nt	AUGUACACGGAGUCGACCCAACGCGA/3Phos/
Marker: 34nt	AUGUACACGGAGUCGAGCUCAACCCGCAACGCGA/3Phos/
Adaptor:	rAppTGGAATTCTCGGGTGCCAAGG/ddC/
RT primer:<br>B is a barcode nucleotide<br>N is a random UMI nucleotide	/5Phos/GGBBBBBNNNNNNNNAGATCGGAAGAGCGTCGTGTAGGGAAAGAGTGT/iSp18/CTCGGCATTCCTGCTGAACCGCTCTTCCGATCTCCTTGGCACCCGAGAATTCCA
Library PCR primer: Forward<br>* indicates a phosphorothioate bond	AATGATACGGCGACCACCGAGATCTACACTCTTTCCCTACACGACGCTCTTCCGATC*T
Library PCR primer: Reverse	CAAGCAGAAGACGGCATACGAGATCGGTCTCGGCATTCCTGCTGAACCGCTCTTCCGATC*T
Mouse/Human shared rRNA depletion probe:<br>Mm-28S-2685-2722	/5BiotinTEG/TTGGGCGCGCGCCGCGGCTGGACGAGGCGCCGCCGCCCT
Mouse rRNA depletion probe:<br>Mm-28S-2927-2933	/5BiotinTEG/AGCGGGCCCCCGGTGGGGCGGGGGGCCCGGACAC
Mouse rRNA depletion probe:<br>Mm-28S-989-1011	/5BiotinTEG/CGGGGCCCGGTGGGGGGCGGGGCGGACTGT
Mouse rRNA depletion probe:<br>Mm-28S-1014-1049	/5BiotinTEG/CCAGTGCGCCCCGGGCGTCGTCGCGCCGTCGGGTCC
Mouse rRNA depletion probe:<br>Mm-28S-1077-1109	/5BiotinTEG/CGACGAAGCCGAGCGCACGGGGTCGGCGGCGAT
rRNA depletion probe:<br>Mm-28S-449-484	/5BiotinTEG/GCAGTCCGCCCGGAGGATTCAACCCGGCGGCGCGCG
Human rRNA depletion probe: Hs-28S-1152-1180	/5BiotinTEG/TGCGCGGGTCGGGGGGCGGGGCGGACTGTC

Human rRNA depletion probe: Hs-28S-484-521	/5BiotinTEG/TCCGGCCGTGTCGGCGGCGGCGGATCTT TCCCGCCCT
Human rRNA depletion probe: Hs-28S-1106-1133	/5BiotinTEG/ACCCCTCCTCCCCGCGCCCCCGCCCCGG
Human rRNA depletion probe: Hs-28S-1409-1442	/5BiotinTEG/TCTCGCCGGCCGAGGTGGGATCCCGAGG CCTCTCC
Human rRNA depletion probe: Hs-28S-1024-1054	/5BiotinTEG/GGGGGGTCTCCCCCGCGGGGGCGCGCCG GCG
